# Unklare chronische Schwindelsyndrome – Erfahrungen mit einem interdisziplinären stationären Diagnostikkonzept

**DOI:** 10.1007/s00106-021-01059-4

**Published:** 2021-05-04

**Authors:** J. Münst, A. Pudszuhn, M. v. Bernstorff, T. Obermueller, H. Erdur, H. J. Audebert, M. Rose, A. Reisshauer, I. Hoffmann, U. Schönfeld, V. M. Hofmann

**Affiliations:** 1 Klinik für Hals-Nasen-Ohrenheilkunde, Campus Benjamin Franklin, Charité – Universitätsmedizin Berlin, corporate member of Freie Universität Berlin, Humboldt-Universität zu Berlin, and Berlin Institute of Health, Hindenburgdamm 30, 12203 Berlin, Deutschland; 2 Klinik und Hochschulambulanz für Neurologie, Campus Benjamin Franklin, Charité – Universitätsmedizin Berlin, corporate member of Freie Universität Berlin, Humboldt-Universität zu Berlin, and Berlin Institute of Health, Berlin, Deutschland; 3 Medizinische Klinik mit Schwerpunkt Psychosomatik, Campus Benjamin Franklin, Charité – Universitätsmedizin Berlin, corporate member of Freie Universität Berlin, Humboldt-Universität zu Berlin, and Berlin Institute of Health, Berlin, Deutschland; 4 Arbeitsbereich Physikalische und Rehabilitative Medizin, Charité – Universitätsmedizin Berlin, corporate member of Freie Universität Berlin, Humboldt-Universität zu Berlin, and Berlin Institute of Health, Berlin, Deutschland

**Keywords:** Neuritis vestibularis, Interdisziplinäre Schwindeldiagnostik, Neuropathia vestibularis, Funktioneller Schwindel, BPLS, Zervikaler Schwindel, Vestibular neuritis, Interdisciplinary vertigo diagnostics, Vestibular neuropathy, Functional vertigo, BPPV, Cervical dizziness

## Abstract

Schwindel ist ein häufiges Leitsymptom. Insbesondere Patienten mit chronischen Schwindelsyndromen erleben eine deutliche Beeinträchtigung der Lebensqualität und bei Berufstätigen eine Einschränkung der Arbeitsfähigkeit. Folgen sind finanzielle und kapazitive Belastungen des Gesundheitssystems aufgrund von häufigen Mehrfachuntersuchungen und Krankschreibungen bis hin zur Erwerbsunfähigkeit der Betroffenen. Bei 150 Patienten mit in der ambulanten Diagnostik unklaren chronischen Schwindelsyndromen wurde auf der Grundlage eines strukturierten interdisziplinären stationären Diagnostikkonzepts bei über 90 % der Fälle mindestens eine die Beschwerden begründende Diagnose erhoben. Chronische Schwindelsyndrome sind häufig multifaktoriell bedingt. Bei mehr als der Hälfte der Patienten fanden sich u. a. psychosomatische (Begleit‑)Diagnosen. Zielführende therapeutische Empfehlungen können nur diagnosespezifisch erfolgen, weshalb in dauerhaft unklaren Fällen auch die Abklärung im Rahmen eines interdisziplinären stationären Diagnostikkonzepts sinnvoll und gerechtfertigt sein kann.

## Abstract (en)

Dizziness is a common leading symptom. Especially patients with chronic vertigo syndromes experience a significant impairment in quality of life up to a limitation of their ability to work in the case of employed persons. The consequences are financial and capacitive burdens on the health system due to frequently multiple examinations and sick leave up to occupational invalidity of the affected patient. In 150 patients with chronic vertigo syndromes and an unclear outpatient diagnosis, at least one diagnosis that justified the complaint was made in over 90% of cases on the basis of a structured interdisciplinary inpatient diagnostic concept. Chronic vertigo syndromes are often multifactorial. Psychosomatic (accompanying) diagnoses were found in more than half of the patients. Targeted therapy can only be recommended after establishing a specific diagnosis. This justifies an interdisciplinary inpatient diagnostic concept for persistently unclear cases.

Schwindel stellt als ein häufiges Leitsymptom und insbesondere bei chronischen Beschwerden nicht selten eine diagnostische und therapeutische Herausforderung dar [14]. Unter Schwindel wird im Deutschen eine „Unsicherheit im Raum“ oder die „fälschliche Wahrnehmung von Bewegungen der Umgebung oder des Körpers“ verstanden.

Die Beschwerden werden im Englischen als „vertigo“, „dizziness“, aber auch beispielsweise als „presyncopal faintness“, „dysequilibrium“ und „nonspecific light-headedness“ beschrieben [2, 14]. Das Komitee der Bárány-Gesellschaft hat 2009 eine internationale Klassifikation der Gleichgewichtserkrankungen (ICVD-1) veröffentlicht, die in erster Ebene eine gemeinsame Definition der primären („vertigo“, „dizziness“, vestibulovisuelle und posturale Symptome) sowie sekundären Symptome beinhaltet [2]. Auf zweiter Ebene werden akute (AVS), episodische und chronische vestibuläre Syndrome (CVS), in dritter Ebene definierte Erkrankungen und in vierter Ebene die zugrunde liegenden Pathomechanismen unterschieden [3]. Schwindel vereint somit einen weitreichenden Symptomkomplex unterschiedlicher Differenzialdiagnosen. Ursachen können sowohl otoneurologische, neurologische, kardiovaskuläre, orthopädische, funktionelle als auch ophthalmologische Erkrankungen sein.

Insgesamt leiden vermutlich 20–30 % der gesamten Weltbevölkerung im Laufe ihres Lebens an Schwindel [7, 14]. Gemäß einer Datenerhebung aus dem Jahr 2015 entspricht dies einer Prävalenz von 6,5 % bezogen auf die gesamte deutsche Bevölkerung (Internationale statistische Klassifikation der Krankheiten, German Modification – ICD-10-GM-2020: H81.- und R42) [8].

In der Diagnostik des AVS sind die meisten Untersucher geübt. Einen besonderen Stellenwert nimmt hier die Testung der HINTS (Head Impulse Test, Nystagmus Test, Test of Skew) bei der Differenzierung zwischen zentralen und peripher-vestibulären Störungen ein [22].

Die Diagnostik des CVS, definiert durch länger als drei Monate anhaltende Schwindelbeschwerden, stellt sich häufig herausfordernder für eine einzelne Fachdisziplin dar. Gerade chronischer Schwindel hat aber einen entscheidenden Einfluss auf die langfristige Lebensqualität durch die Einschränkung der täglichen Aktivitäten und führt zu häufigen Arztbesuchen [17]. In einer deutschen Studie mit insgesamt 4869 Erwachsenen konnte bei Patienten mit vestibulärem Schwindel eine deutliche Einschränkung der geschlechts- und altersadjustierten Lebensqualität im Vergleich zu schwindelfreien Personen beobachtet werden [18]. Insbesondere Patienten mit CVS sind davon betroffen [1, 24]. Zudem leiden diese Patienten häufig an begleitenden psychischen Problemen wie Angstzuständen, Depressionen und Somatisierungsstörungen [29].

Folgen sind finanzielle und kapazitive Belastungen des Gesundheitssystems. Aus einer norwegischen Studie (2008) geht hervor, dass bei einem Anteil von 0,9 % der Frauen und 0,7 % der Männer mit einer Langzeitkrankschreibung (mehr als 8 Wochen) aufgrund von Schwindel 23 % der Frauen und 24 % der Männer im Verlauf eine Berufsunfähigkeitsrente erhielten [23]. Die Mortalität aller Schwindelpatienten ist mit einer Odds Ratio von 1,7 erhöht [4].

Schwindel „unklarer Genese“ stellt mit einer Prävalenz von 4,8 % die häufigste Diagnose (ICD-10-GM-2020 – R42) an Schwindelerkrankungen dar [8]. In diesen Fällen sind insbesondere die therapeutischen Empfehlungen eingeschränkt. Nicht selten stellen sich multimorbide Patienten mit sekundären neurodegenerativen Grunderkrankungen, Patienten mit Polyneuropathien, Sehstörungen und/oder dem dringenden Verdacht auf primären bzw. sekundären funktionellen Schwindel vor. Aus diesem Grund ist, zum besseren Verständnis komplexer CVS und deren Pathophysiologie, eine systematische Diagnostik und Therapie essenziell. Die Differenzialdiagnostik kann durch eine multidisziplinäre Beurteilung, eine sensorspezifische apparative otoneurologische Diagnostik und häufig auch eine Schnittbilddiagnostik gelingen. Idealerweise findet diese Behandlung an einem spezialisierten Zentrum statt, an dem Fachärzte für HNO-Heilkunde, Neurologie, Psychosomatik, physikalische Medizin, Augenheilkunde, diagnostische Radiologie sowie innere Medizin mit den Schwerpunkten psychosomatische Medizin bzw. Kardiologie multidisziplinär zusammenarbeiten. Die dafür geforderte Infrastruktur ist in Deutschland nicht flächendeckend vorhanden, sondern lediglich an einigen (meist hochschulmedizinischen) Zentren bzw. in spezialisierten Praxiszentren implementiert. Die derzeitige Versorgungsrealität im kassenärztlichen Bereich stellt sich für viele Patienten mit unklarem Schwindel als unbefriedigend dar. Ebenso ist für die behandelnden Ärzte der Aufwand für die zeitlich und apparativ anspruchsvolle Patientengruppe nicht adäquat abgebildet. Häufig werden diese Patienten „zur weiteren Behandlung“ aus dem vertragsärztlichen Bereich in die ambulante Hochschulmedizin überwiesen. Auf der Basis dieses Bedarfs wurde an unserer Klinik 2015 ein stationäres interdisziplinäres diagnostisches Konzept etabliert. Der sich über zwei bis drei Tage erstreckende Behandlungsgang bietet, neben einer qualitativ hochwertigen apparativen otoneurologischen und radiologischen Diagnostik, die Möglichkeit einer ggf. auch mehrfachen unmittelbaren Kommunikation von Ärzten unterschiedlicher Fachrichtungen und Hierarchiestufen miteinander, direkt am Patientenbett.

In dieser Studie wird die Bedeutung des entwickelten stationären Diagnostikkonzepts bei Patienten mit unklaren CVS untersucht. Das stationäre Konzept ist explizit für eine spezielle Patientengruppe vorbehalten, bei der während der ambulanten Abklärung keine wegweisende Diagnosestellung gelang.

## Material und Methoden

In einem ausführlichen Gespräch im Rahmen der ambulanten Schwindelsprechstunde wurde das unklare Schwindelsyndrom (wie z. B. Dauer, Häufigkeit, Charakter, Triggerfaktoren usw.) anhand einer durch einen Fragebogen ergänzten standardisierten Anamnese und des Dizziness Handicap Inventory (DHI – German Version, [9, 28]) klassifiziert. Außerdem wurden der Zeitpunkt des Erstereignisses sowie die Begleitsymptome erhoben. Eine Unterscheidung der Begleitsymptome erfolgte in „Ohrsymptome“, „vegetative Symptome“ und „andere Symptome“ (z. B. Zephalgien).

Bei der ambulanten Voruntersuchung erfolgte die Erhebung des vollständigen HNO-Spiegelbefundes. Die klinische Gleichgewichtsuntersuchung mit der Frenzel-Brille umfasste die Prüfung von Spontan‑, Provokations- und Blickrichtungsnystagmen sowie Lage- und Lagerungsnystagmen. Außerdem wurde ein Bedside-Kopfimpulstest zur Beurteilung eines horizontalen Vestibulookulären-Reflex-Defizits durchgeführt sowie das Vorliegen einer Skew-Deviation und horizontaler und/oder vertikaler Blickparesen geprüft. Zudem erfolgten vestibulospinale Prüfungen (Romberg-Steh- und Unterberger-Tretversuch). Aus Kapazitätsgründen wurde im Rahmen der ambulanten Untersuchung an apparativer Diagnostik meist nur ein Reintonaudiogramm und ein Tympanogramm durchgeführt.

Die prästationäre Zusammenschau aller klinischen Befunde, unter Berücksichtigung der andernorts erhobenen Vorbefunde, einschließlich einer Zweitbeurteilung bereits erfolgter radiologischer Bildgebung durch einen spezialisierten Radiologen des Klinikums, ergänzten die Befundung.

**Figure 1:**
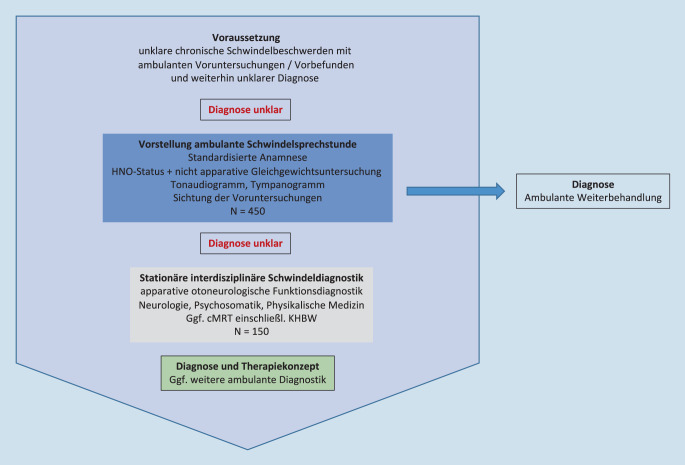

Dabei wurde evaluiert, ob eine Diagnosestellung und ggf. Therapieeinleitung bereits aufgrund der vorhandenen Datenlage möglich waren. Bei weiterhin unklarer Diagnose wurde die Aufnahme zur interdisziplinären stationären Schwindeldiagnostik indiziert (Abb. 1).

Der stationäre Aufenthalt erfolgte für eine Dauer von zwei bis drei Tagen. In diesem Zeitraum wurde die Diagnostik mittels Video-Nystagmographie mit Spontan- und Lagenystagmen, kalorischer Testung der horizontalen Bogengänge, Drehpendelversuch, zervikal und okulär vestibulär evozierter myogener Potenziale (c- und o‑VEMP), Video-Kopfimpulstest (vKIT) aller sechs Bogengänge, okulomotorische Tests (Blickfolge, Blickrichtungen, Sakkadentest, optokinetische Reizung), Halsdrehtest und der subjektiven visuellen Vertikale (SVV) durchgeführt. Standardmäßig erfolgte die Tonschwellenaudiometrie und Impedanzaudiometrie. Sprachaudiometrische Untersuchungen, otoakustische Emissionen, BERA und Elektrocochleographie erfolgten nicht regelhaft, sondern entsprechend der differenzialdiagnostischen Überlegungen. Außerdem wurden die Patienten konsiliarisch standardisiert grundsätzlich neurologisch, psychosomatisch und physikalisch-medizinisch untersucht. Befundabhängig wurden weitere Fachdisziplinen, wie z. B. die Augenheilkunde oder die Radiologie, einbezogen.

In der Untersuchung durch den neurologischen Konsiliararzt erfolgten neben der neurologischen allgemeinen Untersuchung eine ausführliche Testung der Gleichgewichts- bzw. Okulomotorikfunktionen und Tiefensensibilität sowie eine Mitbeurteilung der Anamnese und der prästationären bzw. im Verlauf des Krankenhausaufenthalts angefertigten, zerebralen und ggf. spinalen Bildgebung. Bei klinischen Zeichen einer Polyneuropathie wurde eine elektrophysiologische Untersuchung ergänzt.

Im Rahmen der physikalisch-medizinischen Untersuchung erfolgte nach der Anamneseerhebung ein manualmedizinischer Untersuchungsgang mit dem Ziel, typische Befundkonstellationen, z. B. des „oberen gekreuzten Syndroms“, zu detektieren und dadurch eine gezielte manualtherapeutische Behandlung einzuleiten. Untersucht wurden die klinischen Zeichen der Tiefenstabilität, Muskeltonus sowie häufig beteiligte Schlüsselregionen der Wirbelsäule und der Extremitäten. Dabei wurde zunächst global orientierend (Inspektion und Palpation im Stand) und dann regional orientierend (Wirbelsäule, Becken und Fußgelenke) geprüft. Im weiteren Untersuchungsverlauf erfolgte, je nach Ergebnis der orientierenden Befundung, eine gezielte manualmedizinische Untersuchung wie die segmentale Beweglichkeitsprüfung der Kopfgelenke und Palpation der Schultergürtel- und Kaumuskulatur auf Triggerpunkte (beim Schwindel gehäuft im M. sternocleidomastoideus, M. trapezius pars descendens, M. masseter oder den kleinen Nackenstreckern).

Die psychosomatische Evaluation erfolgte im Liaisondienst als regelhafter Teil der Differenzialdiagnostik in Form eines Erstinterviews über 50 min im Einzelkontakt; abhängig von der Befundlage, ggf. ergänzt durch weitere Gespräche und eine testpsychologische Testung mit standardisierten Fragebögen oder mithilfe eines Strukturierten Klinischen Interviews (SKID-Achse 1). Die Gespräche wurden von approbierten Psycholog/innen der Medizinischen Klinik mit Schwerpunkt Psychosomatik mit mindestens fünf Jahren Berufserfahrung durchgeführt.

Die Diagnosefestlegung erfolgte anhand der erhobenen anamnestischen, klinischen und apparativen interdisziplinären Untersuchungsbefunde und der diagnosespezifischen Kriterien [16]. Dabei wurde die Einordnung der Diagnosen befundabhängig interdisziplinär diskutiert und bzgl. der diagnostischen Sicherheit (Gesichert, Verdacht auf, Zustand nach) und Diagnosehierarchie bewertet.

Die statistische Auswertung der erhobenen Daten erfolgte über IBM SPSS 25. Die Daten wurden mit Häufigkeiten, Mittelwerten, Minima und Maxima einschließlich der Standardabweichung (SD) beschrieben. Die Normalverteilung wurde mittels Kolmogorov-Smirnov-Test bestätigt. Das Signifikanzniveau wurde bei normalverteilten Daten auf a = 0,05 festgelegt und mittels t‑Test sowie χ^2^-Test berechnet.

## Ergebnisse

**Figure 2:**
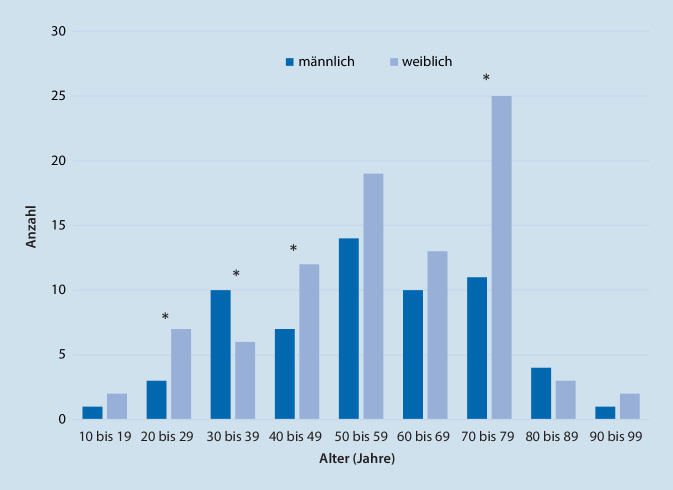

An einer Klinik der Maximalversorgung erfolgte im Zeitraum von Januar bis Dezember 2018 bei über 450 Patienten mit unklarem Schwindel (ICD-10-GM-2020 – R 42) eine Vorstellung in der ambulanten Schwindelsprechstunde. Bei 150 Patienten wurde elektiv ein standardisiertes stationäres Diagnosekonzept (Abb. 1) durchgeführt und retrospektiv ausgewertet. Hierbei waren 89 Patienten weiblich (59,3 %) und 61 Patienten männlich (40,7 %). Der Altersmittelwert der Patienten betrug 57 ± 18 Jahre mit einer Spannweite von 12–92 Jahren. Geschlechtsspezifisch fand sich bei einem Altersmittelwert von 58 Jahren bei Frauen und 56 Jahren bei Männern kein statistisch signifikanter Unterschied (*p* = 0,56; t‑Test). Insgesamt zeigte sich eine Häufung aller Patienten zwischen dem 50. und 79. Lebensjahr, wobei der Unterschied zwischen den Geschlechtern (Frauen > Männer) in der dritten, vierten sowie achten Dekade auf dem Niveau *p* < 0,05 zugunsten der Frauen signifikant war (χ^2^--Test; Abb. 2). In der Altersgruppe der 30- bis 39-Jährigen fand sich isoliert eine signifikante Häufung der Männer.

**Figure 3:**
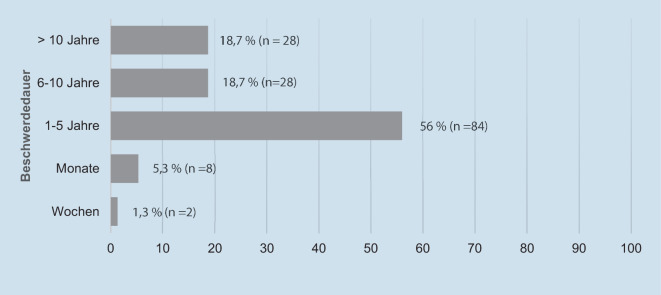

Die Zeitspanne zwischen dem ersten Auftreten von Schwindelsymptomen und der stationären Abklärung betrug in 56 % der Fälle ein bis fünf Jahre. In über 35 % der Fälle litten die Patienten bereits mehr als sechs Jahre unter Schwindelsymptomen. Über ein kürzeres Beschwerdeintervall bis zur stationären Diagnostik berichteten 8 Patienten mit mindestens 3 Monaten und nur 2 Patienten mit mehreren Wochen. (Abb. 3).

### Voruntersuchungen

In 93,3 % (140/150) der Fälle waren Voruntersuchungsbefunde aus der ambulanten Diagnostik vorhanden (Tab. 1). Bei 6,7 % der Patienten gab es keine Vorbefunde bei stationärer Aufnahme.

**Table 1:** 

	Vorbefunde	cMRT	cCT	FKDS	Kalorische Testung	Neurologie	Orthopädie	Innere Medizin	Augenheilkunde
Vorhanden	140 (93,3 %)	105 (70,0 %)	25 (17,2 %)	40 (27,6 %)	51 (34,9 %)	95 (64,6 %)	61 (41,8 %)	66 (44,9 %)	38 (27,1 %)

In 70 % der Fälle (105/150) wurde bereits ambulant eine Magnetresonanztomographie des Kopfs (cMRT) durchgeführt. Bei der Zweitbefundung der cMRT wurde die Bildqualität, bezogen auf die Beurteilbarkeit des Kleinhirnbrückenwinkels und der cochleovestibulären Strukturen, in 9,5 % (10/105) der Fälle als sehr gut mit nachweislich durchgeführter CISS-Sequenz, in 50,5 % (53/105) der Fälle als ausreichend mit einer Schichtdicke von 1,0 mm bewertet. In 21,0 % (22/105) der Fälle wurde die Qualität als nicht ausreichend eingestuft, in 22,9 % (24/105) der Fälle konnte keine weitere Aussage bezüglich der Qualität getroffen werden. In 31,4 % (33/105) der Fälle lag weniger als ein Jahr zwischen der Durchführung der cMRT und dem Zeitpunkt der stationären Untersuchung. In 43,8 % (46/105) der Fälle war die Bildgebung zwischen ein und zwei Jahre alt und in 27,6 % (29/105) der Fälle älter als zwei Jahre. Insgesamt wurde in 36,0 % (54/150) der Fälle eine cMRT-Aufnahme während des stationären Aufenthalts durchgeführt.

In 17,2 % wurden eine ambulante Computertomographie des Schädels (cCT) und des Felsenbeins und in 27,6 % eine farbkodierte Duplexsonographie (FKDS) der Halsgefäße durchgeführt. Ambulant hatten bereits 64,6 % der Patienten eine neurologische, 44,9 % eine internistische und 41,8 % eine orthopädische Abklärung erhalten. Eine augenärztliche Beurteilung war in 25,3 % der Fälle erfolgt. Eine ambulante kalorische Untersuchung der Vestibularorgane (Wasser oder Luft) lag in 34,9 % der Fälle vor.

### Diagnosen

In 94,0 % der Fälle wurde nach der stationären interdisziplinären Diagnostik mindestens eine Ursache für die Schwindelbeschwerden gefunden (Tab. 2). Eine alleinige Diagnose als Grund für die chronischen Schwindelbeschwerden fand sich in 22,7 % (34/150) der Fälle. In 77,3 % (116/150) der Fälle konnten zwei Diagnosen und in 32,7 % (49/150) sogar drei Diagnosen gestellt werden. Vier Diagnosen wurden in 6,7 % (10/150) erhoben. Unklar blieb die Ursache des CVS in nur 6,0 % der Fälle. Generell erfolgte diesbezüglich die Herangehensweise ergebnisoffen. Nach vollständiger Befunderhebung und abgeschlossener apparativer Diagnostik erfolgte interdisziplinär die Festlegung der Diagnosesicherheit und auch der Diagnosehierarchie im direkten Kontakt mit den Behandlern der Fachrichtungen Neurologie, Psychosomatik und physikalische Medizin.

**Table 2:** 

	**Hauptdiagnose** ***n*** **=** **150**	2. Diagnose *n* = 116	3. Diagnose *n* = 49	4. Diagnose *n* = 10	**Schwindelambulanz München [**26**]** ***n*** **=** **34.860**	**Schwindelambulanz Essen, [**20**, **21**]**	
						***n*** **=** **1272**	***n*** **=** **4467**
Jahr der Publikation	**2021**	2021	2021	2021	**2021**	**2015**	**2012**
Periphere Vestibulopathie	**24,0** **% (36)**	8,6 % (10)	4,1 % (2)		**15,8** **%**	**16,0** **%**	**17,4** **%**
– *Unilateral*	**16,7** **% (25)**				**9,1** **%**	**10,5** **%**	**11,3** **%**
– *Bilateral*	**7,3** **% (11)**				**6,7** **%**	**5,5** **%**	**6,1** **%**
Otolithen-Funktionsstörung	**4,0** **% (6)**	4,3 % (5)	4,1 % (2)	–	**–**	**–**	
Benigner paroxysmaler Lagerungsschwindel	**2,7** **% (4)**	4,3 % (5)	–	–	**14,3** **%**	**14,4** **%**	**13,3** **%**
Morbus Menière	**9,3** **% (14)**	3,5 % (4)	–	–	**10,1** **%**	**9,8** **%**	**7,6** **%**
Funktioneller Schwindel	**21,3** **% (32)**	29,1 % (34)	32,7 % (16)	60,0 % (6)	**17,3** **%**	**28,7** **%**	**32,4** **%**
Zentraler Schwindel	**11,4** **% (17)**	11,2 % (13)	6,1 % (3)	–	**13,4** **%**	**8,0** **%**	**7,9** **%**
– *Vestibularisschwannom*	**2,7** **% (4)**	1,7 % (2)	–	–	**–**	**–**	
– *Vertebrobasiläre Insuffizienz*	**0,7** **% (1)**	–	–	–	**–**	**–**	
– *Vestibuläre Übererregbarkeit*	**0,7** **% (1)**	0,9 % (1)	–	–	**–**	**–**	
– *Andere zentral bedingt*	**7,3** **% (11)**						
Vestibuläre Migräne	**4,0** **% (6)**	4,3 % (5)	2,0 % (1)	–	**12,3** **%**	**6,9** **%**	**7,6** **%**
Vestibularisparoxysmie	**0,7** **% (1)**	2,6 % (3)	4,1 % (2)	–	**3,2** **%**	**3,1** **%**	**2,6** **%**
Zervikogener Schwindel	**6,0** **% (9)**	14,6 % (17)	14,3 % (7)	10,0 % (1)	**–**	**–**	
Periphere Polyneuropathie	**9,3** **% (14)**	9,4 % (11)	12,2 % (6)	30,0 % (3)	**–**	**4,2** **%**	
Multifaktorielle Gangstörung	**1,3** **% (2)**	–	–	–	**–**	**–**	
Orthostatischer Schwindel	**–**	–	2,0 % (1)	–	**–**	**–**	
Stenose Karotiden mit V. a. „rotational vertebral artery syndrome“	**–**	0,9 % (1)	2,0 % (1)	–	**–**	**–**	
Dissektion A. carotis interna einseitig	**–**	–	2,0 % (1)	–	**–**	**–**	
Andere^a^	**–**	**4,3 % (5)**	**14,3 % (7)**	–	**8,6** **%**	**–**	
Unklarer Schwindel	**6,0** **% (9)**	–	–	–	**4,5** **%**	**8,7** **%**	**11,2** **%**

^a^Andere Schwindelsyndrome wie z. B. internistische Erkrankungen usw.

Die Einteilung der Diagnosen erfolgte in Hauptdiagnosen, sowie nach abnehmender Relevanz, in Zweit‑, Dritt- und Viertdiagnosen (Tab. 3). Jedoch wurde von den 9 (6 %) unklaren Fällen in 4 Fällen eine 2. Diagnose, in 3 Fällen eine 3. Diagnose neu gefunden, die jedoch nicht hauptursächlich mit dem Schwindel in Zusammenhang gebracht werden konnten.

**Table 3:** 

	Hauptdiagnose (*n* = 150)	2. Diagnose (*n* = 116)	3. Diagnose (*n* = 49)	4. Diagnose (*n* = 10)
Sicher	79 (54,1 %)	52 (44,8 %)	28 (57,1 %)	4 (40,0 %)
Verdacht auf	64 (43,8 %)	62 (53,4 %)	21 (42,9 %)	6 (60,0 %)
Zustand nach	1 (0,7 %)	1 (0,9 %)	–	–
Keine Angabe	2 (1,4 %)	1 (0,9 %)	–	–

Die Hauptdiagnose war in 54,1 % der Fälle sicher, in 43,8 % bestand der Verdacht auf die Erkrankung (Tab. 3). In einem Fall bestand ein Zustand nach der diagnostizierten Erkrankung, in zwei Fällen gab es keine differenzierten Angaben zur Diagnosesicherheit.

Die periphere Vestibulopathie (PVP, Synonyme: Neuropathia vestibularis, Vestibulopathie, Neuritis vestibularis, Neuronitis vestibularis) war in 24,0 % der Fälle die am häufigsten gestellte Hauptdiagnose (Tab. 2). Die PVP konnte in 16,7 % (25/150) unilateral und in 7,3 % (11/150) bilateral nachgewiesen werden. In 21,3 % der Fälle stellte sich die Hauptdiagnose als funktioneller Schwindel dar, d. h. er ließ sich mit psychosozial disponierenden Faktoren oder aktuellen Belastungen in enge Verbindung bringen. In jeweils 9,3 % der Fälle wurde ein Morbus Menière oder eine periphere Polyneuropathie diagnostiziert. Ein benigner paroxysmaler Lagerungschwindel (BPLS) wurde in 2,7 % der Fälle erstdiagnostiziert.

Die Zweitdiagnose zeigte eine Häufung im funktionellen mit 29,1 % (34/116) und im zervikogenen Bereich mit 14,6 % (17/116) (Tab. 2). In 44,8 % war die Zweitdiagnose sicher, in 53,4 % der Fälle wurde eine Verdachtsdiagnose gestellt (Tab. 3).

Die Drittdiagnose war überwiegend dem funktionellen Bereich 32,7 % (16/49) zuzuordnen. In 14,3 % (7/49) wurden jeweils zervikogene Ursachen oder Migränekopfschmerz diagnostiziert (Tab. 2). In 12,2 % (6/49) der Fälle wurde eine Polyneuropathie diagnostiziert. Die Drittdiagnose war in 57,1 % sicher und in 42,9 % ein Verdacht (Tab. 3).

Auch die Viertdiagnose war in 60,0 % (6/10) dem funktionellen Schwindel zuzuordnen (Tab. 2). In 40 % war die Viertdiagnose sicher, in 60 % wurde diese als Verdachtsdiagnose gestellt (Tab. 3).

## Diskussion

### Epidemiologie

Zur Diagnostik unklarer chronischer Schwindelsyndrome wurden Frauen (ca. 2/3) häufiger stationär behandelt als Männer. Vorwiegend ambulant und über Notaufnahmen erhobene Daten an einer großen Population zeigen eine signifikante Häufung der Behandlung von Schwindelbeschwerden bei Frauen in Deutschland [8]. Neuhauser et al. konnten auch eine Einjahresprävalenz für Schwindel mit einem Verhältnis von 1:2,7 der Männer zu Frauen nachweisen [19].

Die Altersverteilung bei unklaren Schwindelsyndromen zeigte in unserer Kohorte einen Altersdurchschnitt von 57 Jahren ohne signifikante Altersdifferenz zwischen den Geschlechtern. Nur ein Drittel der Patienten war dabei ≥ 67 Jahre alt. Aus einer aktuellen deutschen Datenanalyse geht hervor, dass im Besonderen die Diagnose „Schwindel unklarer Genese“ (R42) deutschlandweit eine Häufung im Alter zwischen 85 und 89 Jahren hat [8]. Mutmaßlich liegt insbesondere bei dieser Patientengruppe mit zunächst „unklarem Schwindel“ ein „komplexes Schwindelsyndrom“ vor, das sich durch die Addition von Teildefiziten im visuellen, vestibulären sowie muskuloskeletalen Bereich zu einem erheblichen Gesamtdefizit addiert. Die subjektive bzw. klinisch messbare Ausprägung könnte sich dann nach individuellem Verteilungsmuster unterscheiden. Eine Erklärung für das niedrigere Durchschnittsalter in der vorliegenden Studie könnte der höhere Leidensdruck berufstätiger Patienten in jüngeren Altersgruppen sein. Insbesondere Berufstätige drängen auf eine weitere Abklärung ihrer Symptome, trotz bereits erfolgter ambulanter Diagnostik, da sich die allgemeine Mobilitätseinschränkung und Arbeitsunfähigkeit dauerhaft auf die Erwerbsfähigkeit und den Alltag auswirken. Dabei werden häufig aktiv digitale Medien (Internet) eingesetzt, um spezialisierte Einrichtungen zu finden. Bei Patienten in einem höheren Lebensalter sind oft nicht in der Lage, ihren persistierenden Beschwerden und Einschränkungen ausreichend Ausdruck zu verleihen und nach alternativen Diagnostikmöglichkeiten zu suchen. Möglicherweise wird diese Patientengruppe deshalb in geringerer Zahl dieser interdisziplinären Diagnostik zugeführt. Zudem ist zu vermuten, dass Patienten in einem Alter oberhalb der achten Dekade möglicherweise zum Teil ihren Zustand als „altersentsprechend“ physiologisch und in der Selbstwahrnehmung gar nicht als pathologisch wahrnehmen. Im Sinne eines therapeutischen Nihilismus, gepaart mit einer reduzierten Motivation, wird von einer ärztlichen Konsultation möglicherweise Abstand genommen. Um diesen Punkt zu klären, müsste eine zufällig ausgewählte Bevölkerungsstichprobe bezüglich ihrer Selbstwahrnehmung befragt werden.

Insgesamt lässt sich an den erhobenen Daten über die Zeitspanne zwischen dem ersten Auftreten von Schwindelsymptomen und der stationären Abklärung erkennen, dass über 90 % der Patienten seit mehr als einem Jahr Beschwerden hatten und über 1/3 der Fälle (37,4 %) bereits seit mehr als 6 Jahren unter Schwindelbeschwerden litten, bevor die stationäre interdisziplinäre Schwindelabklärung erfolgte, was das Ausmaß der Chronizität unterstreicht. Angaben über Zeitspannen bis zur Diagnosestellung bei CVS finden sich in der Literatur nicht.

### Voruntersuchungen

Der überwiegende Anteil (91,2 %) der Patienten mit Abklärung im Rahmen der interdisziplinären stationären Schwindeldiagnostik hatte, trotz ambulanter Vorbefunde, wie cMRT, cCT, FKDS sowie in vielen Fällen auch neurologischen, orthopädischen und internistischen Vorstellungen sowie partiell otoneurologischen Untersuchungen, keine befriedigende Einordnung der Beschwerden erfahren.

In Deutschland bekommen durchschnittlich 3,2 % aller Schwindelpatienten eine apparative Diagnostik. Hierbei waren jüngere Patienten deutlich häufiger vertreten als Ältere [6]. Allgemein zeigte sich, dass die bildgebende Diagnostik die am häufigsten durchgeführte und kostenintensivste Methode darstellt [13]. Speziell „Schwindelpatienten“ konsultieren in 61,3 % der Fälle mehr als 2‑mal einen Arzt [6]. Diese Rate steigt bei Vorhandensein einer Angststörung auf 6,6 Arztbesuche innerhalb eines Jahres an [29]. Im Vergleich hierzu lag in der vorliegenden Untersuchung in der Mehrzahl der Fälle (70,0 %) eine cMRT-Aufnahme vor. Dies entspricht dem Bild eines überwiegend gut diagnostizierten Großstadt-Patientenkollektivs. Einerseits ist es das Ziel, Doppeluntersuchungen zu vermeiden. Andererseits muss die Bildgebung hinsichtlich ihrer Untersuchungsqualität (Schichtdicke, Sequenzen) und des Durchführungszeitpunkts in Bezug auf die Symptomatik kritisch beurteilt werden, um Fehlinterpretationen zu vermeiden. In 54/150 (36 %) Fällen wurde eine cMRT-Aufnahme während des stationären Aufenthalts durchgeführt. Davon erhielten 20/105 (19,0 %) Patienten eine erneute cMRT-Aufnahme, obwohl bereits eine ambulant durchgeführt cMRT-Aufnahme vorlag, weil die ambulant durchgeführte Bildgebung den genannten Kriterien zur Untersuchungsqualität bzw. -Zeitpunkt, bezogen auf die Fragestellung, nicht entsprach.

Eine cCT bzw. Felsenbein-CT erfolgte nicht regelhaft im Rahmen der stationären Diagnostik. Eine bereits ambulant im Vorfeld durchgeführte CT-Diagnostik (17 % der Fälle) wurde grundsätzlich vom klinikeigenen Radiologen demonstriert. Dies betraf Patienten, bei denen neben Schwindelbeschwerden eine „Ohranamnese“ oder entsprechende. Symptome vorlagen, um knöcherne Malformationen wie einen erweiterten Aquaeductus vestibuli oder eine Dehiszenz der Bogengänge bzw. voroperierte Veränderungen als Schwindelursache zu beurteilen. Diese Schwindelursachen sind deshalb in unserer Studie nicht bei allen Patienten auszuschließen.

Die vorliegenden Daten, mit der in 30 % der Fälle bereits ambulant durchgeführten kalorischen Testung, zeigen eine im Vergleich mit der Literatur bereits relativ hohe apparative Diagnostikhäufigkeit. Bei 24,0 % (36/150) der Patienten wurde während der stationären Diagnostik die Hauptdiagnose PVP als Ursache für die Schwindelbeschwerden festgestellt. Davon war in 60,7 % der Fälle eine PVP bisher nicht bekannt.

Die ambulante Diagnosehäufigkeit für eine vestibuläre Erkrankung liegt in der Literatur bei durchschnittlich 12,2–13,8 % [5, 6]. Der vKIT wurde ambulant beispielweise nur in 5 % der Fälle durchgeführt, obwohl die höchste Diagnosesicherheit für vestibuläre Erkrankungen beim vKIT liegt [13]. Ambulant sind außerhalb von spezialisierten Zentren und Praxen apparative otoneurologische Untersuchungen, wie der vKIT oder die kalorische Prüfung in der Primärdiagnostik zur Differenzierung von zentralen und peripher-vestibulären Störungen möglicherweise unterrepräsentiert. Ambulante Daten sowohl zur Primärdiagnostik als auch zur erweiterten otoneurologischen Diagnostik (vKIT in allen Ebenen, c‑/o-VEMP, SVV, Drehpendelprüfung) konnten nicht erhoben werden.

In der Literatur ist der Nutzen einer präzisen klinischen und otoneurologischen Primärdiagnostik beim AVS am Beispiel der HINTS-Testung gut nachgewiesen. Hierbei lagen die Sensitivität und Spezifität eines positiven Tests bei 96–100 % bzw. 72–96 % im Hinblick auf den Nachweis eines akuten Infarkts [10, 22]. Wohingegen der Anteil an falsch-negativen Ergebnissen einer cMRT in den ersten 48 h nach initialer Symptomatik bei 12 % lag. Beim AVS konnte außerdem gezeigt werden, dass die Verdachtsdiagnose einer akuten unilateralen PVP nach der klinischen Primäruntersuchung in einer Notaufnahme in der apparativen Diagnostik nur in 69 % der Fälle bestätigt wurde [22]. Eine bereits bei AVS durchgeführte apparative otoneurologische Diagnostik zur Diagnosesicherung ist nicht nur zum Ausschluss eines Schlaganfalls erforderlich, sondern auch zur Findung einer gesicherten Diagnose. Ungeklärte AVS rufen bei Patienten oft Unsicherheit und Ängste hervor, die im Zusammenhang mit einer Chronifizierung der Beschwerden im Sinne eines CVS zu primären oder sekundären somatoformen Störungen führen können [29].

### Diagnosen

Bei 94 % der unklaren CVS konnte mindestens eine den Schwindel verursachende Diagnose definiert werden. Die CVS waren nur in rund 20 % der Fälle durch eine alleinige Diagnose verursacht. Vielmehr zeigte sich in ca. 80 % der Fälle ein multifaktorielles Geschehen, das eine Chronifizierung durch zusätzliche funktionelle Beschwerden begünstigt.

Die Ergebnisse der Diagnosehäufigkeiten und des Diagnosespektrums der stationären Patienten in Berlin (*n* = 150, HNO-Heilkunde) unterscheiden sich teilweise von den ambulanten Daten einer großen Schwindelambulanz in München (*n* = 34.860, Neurologie, [27]; Tab. 2) sowie in Essen (*n* = 4467 und *n* = 1272, Neurologie, [20, 21]). Neben der Populationsgröße ist die interdisziplinäre Schwindeldiagnostik möglicherweise von einer spezifischen Zuweisung durch verschiedene Facharztrichtungen geprägt. Ein Bias aufgrund einer fachspezifischen Zuweisung ist somit in beiden Fällen nicht auszuschließen und muss bei der Interpretation von Häufigkeiten einzelner Erkrankungsentitäten berücksichtigt werden. Ebenso kann die stationäre Ausrichtung des Konzepts das Diagnosenspektrum a priori verändern. In unsere Studie wurden 150 meist seit Jahren schwer betroffene Patienten eingeschlossen, bei denen am Ende in 9,3 % der Fälle die Hauptdiagnose einer Polyneuropathie stand. Diese Diagnose wird in der Münchner Studie nicht aufgeführt und im Kollektiv des integrierten Versorgungskonzepts in Essen (DiVeR-Studie) nur bei 4,2 % der Patienten gefunden [21]. Dieses Ergebnis könnte dadurch erklärt sein, dass bei unserem stationären Konzept bei den Patienten eine im Durchschnitt höhere Komorbidität bzw. Immobilität vorlag. Nicht alle von uns behandelten Patienten eignen sich für ein ambulantes oder teilstationäres Konzept mit täglich notwendigen An- und Abreisen.

In der vorliegenden Studie wurde am häufigsten als Hauptdiagnose eine PVP mit 24 % diagnostiziert. Diese konnte in 2/3 der Fälle unilateral (UPVP) und in 1/3 der Fälle bilateral (BPVP) nachgewiesen werden. Im Vergleich dazu zeigen die Ergebnisse der ambulanten Schwindelambulanz München eine UPVP bei 9,1 % und eine BPVP 6,7 % der Fälle [27] sowie in der Essener Schwindelambulanz eine UPVP bei 17,4 % und eine BPVP in 11,3 % [20, 21]. In allen Studiengruppen findet sich ein deutliches Überwiegen der UPVP. Des Weiteren beschreiben Strupp et al., dass 20–30 % der akuten UPVP nach 12 Monaten weiterhin Schwindelbeschwerden haben, wobei in 70 % der Fälle zu diesem Zeitpunkt weiterhin eine kalorische Untererregbarkeit messbar ist [26]. Aus diesem Grund ist es bedeutsam, die Patienten mit einer schlechteren Kompensation zeitnah zu identifizieren und Komorbiditäten zu diagnostizieren, die eine Kompensation erschweren.

Unsere Arbeit stellt die isolierte Otolithenfunktionsstörung als eigenständige Untergruppe heraus, was sich so in den Münchner und Essener Daten nicht finden lässt. Otolithenfunktionsstörungen zeigen anamnestisch typische Symptome bei Linearbeschleunigungsreizen und können mit c‑ bzw. o‑VEMP-Untersuchungen und dem SVV-Test apparativ erfasst werden. In den Publikationen der beiden Arbeitsgruppen sind isolierte Otolithenfunktionsstörungen nicht separat ausgewiesen [20, 21, 27].

In der vorliegenden Studie stellte der funktionelle Schwindel mit 21,3 % der Patienten die zweithäufigste Hauptdiagnose und in mehr als 37,3 % (56/150) eine der 2.–4. Diagnosen dar. Funktionelle Schwindelsyndrome waren in der Schwindelambulanz München mit 17,3 % sowie der Essener Schwindelambulanz in 28,7 % und 32,4 % der Patienten die häufigsten Diagnosen [20, 21, 27]. Die funktionellen Schwindelbeschwerden wurden in den vergangenen Jahren neu bewertet. Beispielsweise gilt die „persistent postural-perceptual dizziness“ heute als funktionelle vestibuläre Erkrankung, die weder allein struktureller noch allein psychosozialer Genese ist, aber mit anderen psychischen Erkrankungen, wie z. B. Angststörungen oder Depressionen, einhergehen kann [25]. Weitere, dem funktionellen Schwindel zugehörige Krankheitsbilder stellen „phobic postural vertigo“, „visual vestibular mismatch“, „space and motion discomfort“ und „chronic subjective dizziness“ dar. Diese Schwindelsyndrome gehen ebenfalls mit einer Veränderung der vestibulären Funktionen sowie Störungen zentraler und peripherer Ausgleichsmechanismen einher. Die aktuelle Vorstellung ist, dass zwischen den strukturell und funktionell bedingten Schwindelsyndromen fließende Übergänge bestehen bzw. eine Koexistenz unterschiedlicher Krankheitsbilder vorhanden sein kann [25]. Schwindel als funktionelle Störung wird als Ausdruck einer misslingenden Anpassungsfähigkeit basierend auf den individuellen biologischen, psychischen und sozialen Ressourcen an die psychosozialen oder auch biologischen Anforderungen gesehen. Somit führen bei einer vulnerablen biologischen Disposition bereits geringere Belastungen zu Symptomen. Funktionelle Symptome werden daher in der Regel vor dem Hintergrund der Interaktion zwischen Person und Umwelt verstanden.

Der BPLS wurde in dieser Studie nur in 2,7 % der Fälle als Hauptdiagnose gestellt, jedoch in den Schwindelambulanzen München mit 14,3 % respektive in Essen mit 13,3 bzw. 14,4 % der Patienten am zweithäufigsten diagnostiziert [20, 21, 27]. Aus der Literatur ist bekannt, dass bei 25–50 % der Patienten mit BPLS in den ersten 3 Jahren Rezidive auftreten [17]. Bei den CVS der Patienten der vorliegenden Studie ist der BPLS deutlich unterrepräsentiert. Das lässt vermuten, dass in der Mehrzahl der Fälle dieses Krankheitsbild in der ambulanten Diagnostik ausreichend diagnostiziert und therapiert wird.

In der vorliegenden Studie war in einer Gesamtanzahl von 11,4 % der Fälle die Diagnose dem „zentral vestibulären Schwindel“ (Tab. 2, 17/150) zuzuordnen. Die zentral-vestibuläre Störung wird dabei als Überbegriff verwendet, der unterschiedliche Ursachen vereinigt. Ein vergleichbares Ergebnis geht aus den Daten der Schwindelambulanzen München und Essen mit 13,2 bzw. 8 % der Fälle hervor [20, 21, 27].

In unserer Studie war der zervikogene Schwindel mit 6,0 % eine der selteneren Hauptdiagnosen, aber mit zwischen 10–14,5 % eine der häufigsten 2.–4. Diagnosen. Der zervikogene Schwindel ist eine viel diskutierte Diagnose [15, 30] und aus Mangel an eindeutigen objektivierbaren diagnostischen Kriterien oft eine klassische Ausschlussdiagnose [12]. Die manualmedizinische Untersuchung durch die „physikalische Medizin“ fand in unserer Studie standardisiert bei allen Patienten statt. Neben dem standardisierten Untersuchungsgang fand der, im Rahmen der Gleichgewichtsuntersuchung durchgeführte, Halsdrehtest Eingang in die Bewertung. Inwiefern eine gefundene Pathologie als schwindelbegründend eingeschätzt werden konnte, wurde im direkten konsiliarischen Gespräch zwischen den Fachdisziplinen festgelegt.

Unklare CVS nach interdisziplinärer Schwindeldiagnostik finden sich in nur 6 % (Tab. 2). Somit können in über 90 % der unklaren Schwindelsyndrome eine Diagnose gefunden werden und damit diagnosespezifische, teilweise multidisziplinäre Therapieansätze eingeleitet werden.

Die Ergebnisse der Studie zeigen auch, dass die diagnostische Gewissheit bei chronischen Schwindelpatienten in mehr als 50 % als sicher definiert wurde (Tab. 3). Diagnosen werden als Verdachtsdiagnosen bezeichnet, wenn sie am Ende eines stationären Aufenthalts weder sicher bestätigt werden können noch sicher ausgeschlossen sind [11]. So haben Verdachtsdiagnosen einen Einfluss auf die Kodierung (bei Entlassung nach Hause bzw. Verlegung in ein anderes Krankenhaus) und weisen aber auch auf die Bedeutung der anamnesegestützten Diagnosen bei CVS hin. Insbesondere der dokumentierte klare Ausschluss von Diagnosen struktureller Natur kann die Therapie von psychofunktionellen Erkrankungen unterstützen.

Kovacs et al. zeigten in einer Metaanalyse, dass Schwindelbeschwerden generell in über 60 % der Fälle zu mehrfachen Arztkonsultationen führen und diese mit steigendem Alter weiter zunehmen. Zudem wurde gezeigt, dass eine Kostensteigerung durch doppelte und unnötige Untersuchungen vorangetrieben wird. Dies ist vorwiegend in einer fehlenden Koordination der einzelnen Fachdisziplinen und der unzureichenden interdisziplinären Zusammenarbeit begründet [13].

In der vorliegenden Studie ist davon auszugehen, dass zwei Drittel der Patienten (100/150 Patienten) zum Untersuchungszeitpunkt in einem potenziell berufstätigen Alter (< 67 Jahre) waren. Somit stellen neben den Wiederholungsuntersuchungen entstehende Komorbiditäten durch reduzierte Mobilität, Sturzgefährdung und insbesondere die Langzeitarbeitsunfähigkeit zusätzlich kostenrelevante Faktoren für das allgemeine Gesundheitswesen dar.

Zudem beklagen die Patienten eine dauerhaft eingeschränkte krankheitsbezogene Lebensqualität.

Für sich genommen ist es möglich, jeden der beschriebenen anamnestischen, diagnostischen und apparativ-diagnostischen Schritte ambulant durchzuführen, wie es die Konzepte aus den Zentren in München und Essen eindrucksvoll zeigen. Zusammenfassend lassen sich Vorteile einer interdisziplinären stationären Schwindeldiagnostik gegenüber der ambulanten Diagnostik bei einer Untergruppe von schwer betroffenen, reduziert mobilen oder durch Komorbiditäten belasteten Patientengruppe finden. Die mehrfache metachrone Untersuchung und die unmittelbare Zusammenführung aller Befunde sowie die interdisziplinäre Zusammenarbeit der verschiedenen Fachrichtungen, im Sinne eines „echten Konsiliums“ bei diesen Problemfällen, führt zu einer gesamtheitlichen Wahrnehmung des Patienten. Die allermeisten „unklaren Schwindelsyndrome“ bleiben nicht unklar. Die bereits durch die chronische Gleichgewichtsstörung physisch (Gangstörungen, Sturzgefährdung) und psychisch kompromittierten, aber auch insbesondere durch die otoneurologische Testbatterie zusätzlich belasteten Patienten profitieren im Besonderen von dem stationären Behandlungskonzept. Das stationäre Vorgehen ermöglicht dem Untersucher jederzeit, akute Beschwerden des Patienten zeitnah zu beurteilen. Zudem erfolgt ein intensiver direkter Austausch von Informationen zwischen den unterschiedlichen Fachrichtungen, welcher im üblichen hochschulambulanten Untersuchungsgang, aufgrund struktureller Gründe, so nicht realistisch durchführbar ist. Die kritische Einbeziehung der Vorbefunde reduziert einerseits eine Doppeldiagnostik, detektiert aber auch falsch-negative Befunde. In 2,7 % (4/150) der Fälle konnte die Hauptdiagnose eines Vestibularisschwannoms gestellt werden. In allen Fällen wurde bereits eine cMRT-Aufnahme ambulant durchgeführt, wobei in zwei Fällen die Erstdiagnose des Vestibularisschwannoms erst während der stationären Schwindeldiagnostik gesichert werden konnte.

Das stationäre Schwindeldiagnostikkonzept hat in dieser selektierten Patientenkohorte mit CVS und seit Jahren bestehenden Beschwerden erfolgreich zu einer Diagnosefindung beigetragen. Inwieweit eine diagnosespezifische Therapie eine Verbesserung der gesundheitsbezogenen Lebensqualität erwarten lässt und damit Folgekosten für das Gesundheitssystem reduziert werden, bleibt zukünftigen Studien vorbehalten. Dieses Konzept hat auch hinsichtlich der studentischen Lehre und Assistentenausbildung eine besondere didaktische Bedeutung an unserer Hochschule erlangt.

## Fazit

- Bei Patienten mit chronischen Schwindelsyndromen (CVS) gelingt in bestimmten Fällen gegenwärtig keine definitive Diagnosestellung in den ambulanten Versorgungsstrukturen.
- Patienten mit CVS haben bei Vorstellung in über 90 % der Fälle eine bereits mehrjährige Beeinträchtigung ihrer Lebensqualität durch die Schwindelsymptomatik.
- Ein stationäres interdisziplinäres Schwindeldiagnostikkonzept kann durch eine differenzierte otoneurologische Diagnostik und eine ungehinderte Kommunikation der beteiligten Fachdisziplinen auch bei unklaren CVS in mehr als 90 % der Fälle Ursachen identifizieren und die Patienten einer diagnosespezifischen Therapie zuführen.
- Interdisziplinäre Abklärungen von CVS finden bei Frauen häufiger statt als bei Männern.
- Chronische Schwindelsyndrome sind in der Mehrheit der Fälle multifaktoriell bedingt und erfordern diagnoseabhängig auch ein komplexes therapeutisches Vorgehen.
- Bei über der Hälfte der Patienten mit CVS liegt eine psychosomatische (Ko‑)Morbidität vor.

## Abkürzungen

AVS: Akutes vestibuläres Syndrom
BPLS: Benigner paroxysmaler Lagerungsschwindel
BPVP: Bilaterale periphere Vestibulopathie
cCT: Kraniale Computertomographie
CISS: Constructive-Interference-in-Steady-State
cMRT: Kraniale Magnetresonanztomographie
c-/o-VEMP: Zervikal/okulär vestibulär evozierte myogene Potenziale
CVS: Chronisches vestibuläres Syndrom
FKDS: Farbkodierte Duplexsonographie
HINTS: Head Impulse Test, Nystagmus, Test of Skew
SVV: Subjektive visuelle Vertikale
UPVP: Unilaterale periphere Vestibulopathie
v‑KIT: Video-Kopfimpulstest
VOR: Vestibulookulärer Reflex
